# Supplementary material for: Tributyrin alleviates gut microbiota dysbiosis to repair intestinal damage in antibiotic-treated mice
Source: PLoS One. 2023 Jul 31;18(7):e0289364. doi: 10.1371/journal.pone.0289364 (PMC10389721; doi:10.1371/journal.pone.0289364)
Supplement: S1 Raw images — (PDF) [file pone.0289364.s002.pdf]

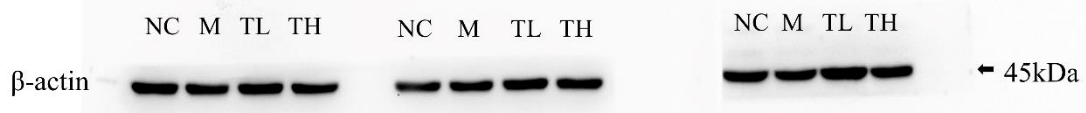

Protein bands were detected by VILBER Fusion FX7 and analyzed by ImageJ.

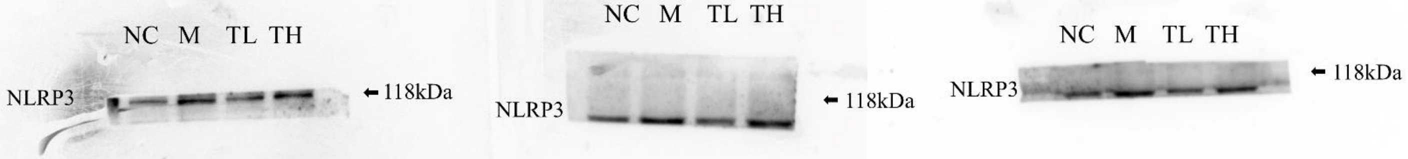

Protein bands were detected by VILBER Fusion FX7 and analyzed by ImageJ.

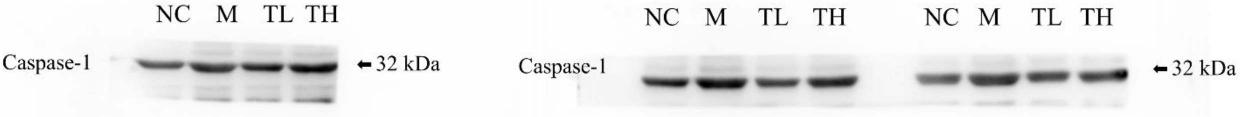

Protein bands were detected by VILBER Fusion FX7 and analyzed by ImageJ.

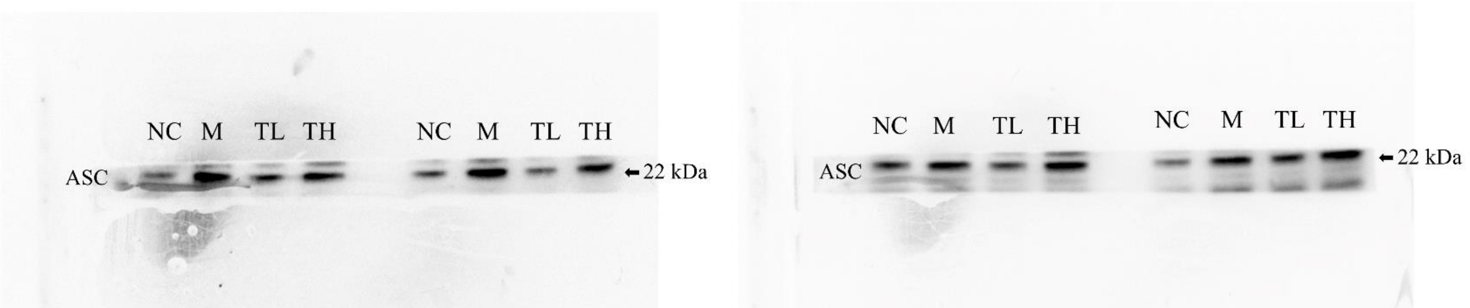

Protein bands were detected by VILBER Fusion FX7 and analyzed by ImageJ.

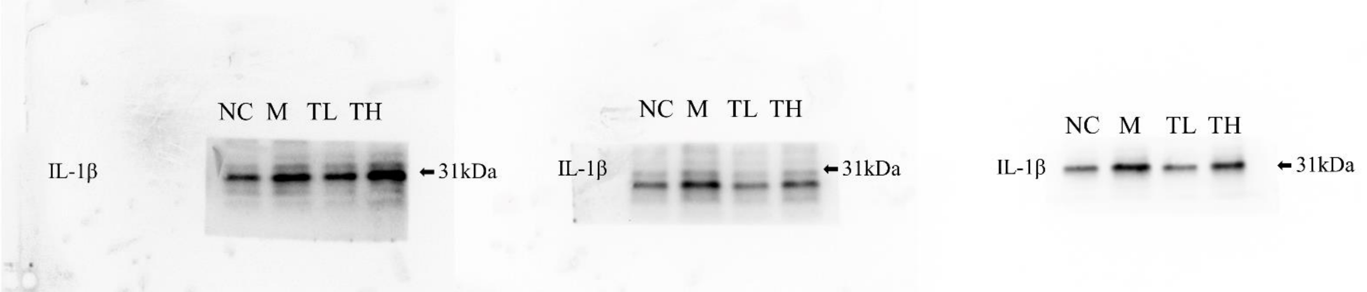

Protein bands were detected by VILBER Fusion FX7 and analyzed by ImageJ.

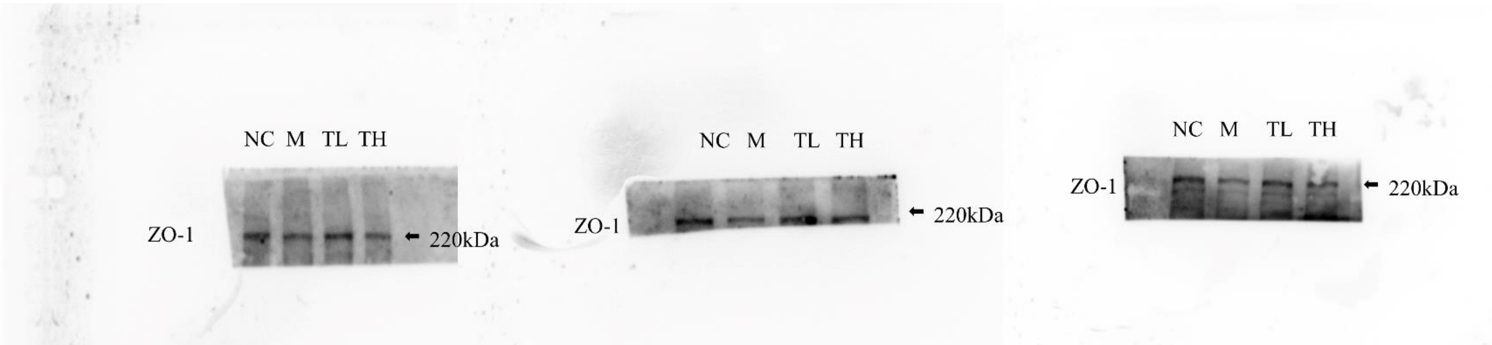

Protein bands were detected by VILBER Fusion FX7 and analyzed by ImageJ.

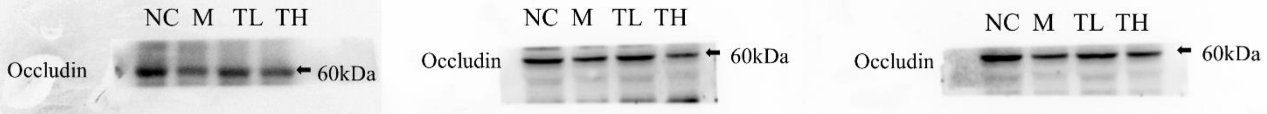

Protein bands were detected by VILBER Fusion FX7 and analyzed by ImageJ.
